# Professionally- and Self-Trained Service Dogs: Benefits and Challenges for Partners With Disabilities

**DOI:** 10.3389/fvets.2019.00179

**Published:** 2019-06-11

**Authors:** Mariko Yamamoto, Lynette A. Hart

**Affiliations:** ^1^Department of Animal Sciences, Teikyo University of Science, Yamanashi, Japan; ^2^School of Veterinary Medicine, University of California, Davis, Davis, CA, United States

**Keywords:** service dogs, self-trained, professionally-trained, family members, mobility, medical, psychiatric

## Abstract

It has been widely reported that service dogs offer benefits to their human partners, however, it is unclear whether the expanding methods of training and roles of service dogs for their partners with various disabilities also provide similar benefits. This study aimed to investigate the self-reported experience of service dog partners to understand whether three different factors influence the benefits and drawbacks associated with partnering with a service dog: (1) different methods of training service dogs; (2) different severities of human partners' disabilities; (3) different roles of service dogs. Partners of service dogs were recruited to the web survey through service dog facilities and networking groups. Answers from 19 men and 147 women participants (91.8% living in the U.S.) were analyzed in this study. Participants experienced the expected benefits of service dogs, including increased independence, social relationships, self-esteem, and life satisfaction, and decreased anxiety, stress, and loneliness. However, the perceived benefits, concerns, and burdens differed depending on the partners' disabilities and the training history of the dogs. When first living with their service dogs, people who had self-trained their service dogs experienced more burdens than those living with professionally trained service dogs. No major reduction in expenses for assistance after acquiring a dog was reported. Personalized team training based on each person's disabilities and situation is required to optimize the benefits and minimize the burdens and concerns of living with service dogs.

## Introduction

Assistance dogs are now frequently seen working in the U.S., where the U.S. Department of Justice uses “service dogs” or “service animals” as the inclusive term (1). However, internationally, “assistance dogs” is the inclusive term used by Assistance Dogs International (ADI) (2): a general term for dogs that support their human partners with various disabilities. The three major ADI classifications of assistance dogs are: guide dogs for people with visual disabilities, hearing dogs for people with hearing disabilities, and service dogs for people with any disabilities other than visual or hearing disabilities (2). Various types of service dogs perform tasks to support people with many different disabilities. After Bonita Bergin first demonstrated the concept of service dogs for mobility support in 1975, new roles were created for service dogs, such as service dogs for people with psychiatric disorders or autism, and for detection of seizures associated with epilepsy or hypoglycemia with diabetes. Prior to 1980, roles of assistance dogs were primarily guide dogs, hearing dogs, and service dogs for mobility support. The abovementioned new roles of service dogs expanded widely especially after 2001 (3). Therefore, in this paper “traditional assistance dogs” or “traditional assistance dog training facilities” are used to indicate types of assistance dogs which roles had been already established prior to 1980 or training facilities which train such assistance dogs. Service dogs with new roles are referred to here as “new roles/types of service dogs.”

Beneficial effects of assistance dogs have been reported for the human partners with disabilities, including increased independence, frequency of leaving the house, and social interactions, as well as decreased paid and unpaid assistance (4–10). Acquisition of assistance dogs also leads to psychological benefits for the partners, such as increased self-esteem and confidence, and decreased anxiety and stress (5, 8, 10). Members of the public commonly understand there are benefits from assistance dogs for their partners, and the popularity of assistance dogs is growing (11). However, the current permissive U.S. situation with assistance dogs raises concerns about too readily assuming that the effects of assistance dogs are inevitably beneficial.

The above mentioned benefits of assistance dogs, especially with guide dogs and service dogs, were usually documented in studies focusing on dogs which had been trained by the traditional assistance dog training facilities (4, 5, 9, 10); usually these were puppies of Labrador or Golden Retrievers or mixed breeds of Labrador and Golden Retrievers. These facilities place the puppies with volunteer caretakers for the first 1–2 years, and then train the dogs at the facility for several months to a year. Persons with disabilities are then assigned dogs and given team training to teach the partners strategies for living with the dogs.

The U. S. has no governmental or federal system for registration or qualification of assistance dogs, nor are any required procedures or certain facilities specified for training of the dogs (1, 12, 13). Further, under the U.S. Code Title 42, disabilities are very broadly defined to offer maximal “reasonable accommodation” to those having disabilities (14), while also protecting the person's privacy regarding the disabilities. This legal context of U.S. laws and regulations means that people can create their personalized assistance dogs as they choose, without any assessment of their dogs' quality or the person's eligibility to be partnered with an assistance dog. This means that assistance dogs in the U.S. sharply differ from each other, having many types of training histories and other characteristics: specific assisting tasks, and sources, sizes, and breeds of the dogs (3). The severity of the partners' disabilities also varies and some people have multiple disabilities, which often change over their life course. Further, increasingly people in the U.S. train their own assistance dogs, especially service dogs (3, 11). It is unknown whether the previously reported benefits from traditional assistance dogs are similar with the new types of service dogs.

This research aimed to investigate the self-reported experiences of service dog partners to understand whether three different factors influence the benefits and drawbacks associated with being partnered with a service dog: (1) methods of training of dogs: self-trained by the partners, or professionally trained by service dog facilities or private trainers; (2) severities of disabilities among partners living with service dogs for mobility assistance: slight/independent, moderate, or severe mobility disabilities; (3) roles/types of assistance by service dogs: mobility, psychiatric or medical assistance. These three factors were selected because they had not previously been well-studied.

Partnering with a service dog does not always improve life for a person with disability. The outcomes are inconsistent among different pairs of service dogs and their human partners. The dogs and humans are both living creatures and the interaction of the two develops into a unique relationship which can have both good and bad aspects. Predicting the outcomes of partnering a service dog has become more difficult than before as new roles of service dogs have been created and these new types of service dogs are little-studied. However, studying currently working service dogs and their partners may provide useful information to gain a better understanding on the relationship of service dogs and their partners and minimize the possible problems.

Persons whose disabilities were most severe were expected to be less likely to embark on self-training a service dog. Self-training seemed likely to pose greater challenges for the partner in achieving useful service support from the dog. Thus, persons electing to train their own dogs, either alone or with assistance from a trainer, were hypothesized to differ in their profiles of disabilities, demographic traits and their experiences of benefits and challenges with the dogs.

## Methods

### Subject and Data Collection

This study focused only on service dog partners; people living with guide dogs and hearing dogs were not included. Guide dogs and hearing dogs were not included in this study because a considerable research literature on them already exists, however there is scarce information on the expanding roles of service dogs. In addition, while the assisting tasks of guide dogs and hearing dogs are fairly conventional and consistent, the assisting tasks of service dogs vary and depend on the particular disabilities of their human partners. The partners' range of disabilities included: mobility disabilities, such as using a wheelchair; psychiatric disorders, such as living with posttraumatic stress disorder (PTSD) and anxiety; and medically related disabilities, including diabetes and epilepsy.

This study was conducted using an online web survey. For recruitment, the study announcement was sent to service dog partners through the International Association of Assistance Dog Partners, service dog training facilities, and social networking groups related to service dog partners. Participation was anonymous and voluntary, and was approved by the University of California, Davis, Institutional Review Board Protocol #340095-2.

### Questionnaire

The questionnaire included standardized surveys to assess the participants' physical and psychological health, physical activity, and level of independence. Additional questions concerned participants' demographic details and their experiences with their service dogs.

1. Physical and Mental Health, Physical Activity, and Independence
  The participants' physical and mental health status was assessed using the Medical Outcomes Study 36-Item Short-Form Health Survey (SF-36), which is a widely used measure of health-related quality of life (15). SF-36 has 36 questions and provides physical and mental health summary scores and does not specify whether the respondents have disabilities or not, nor does it address types of disabilities. It uses norm-based scoring algorithms for the physical and mental health summary scores (T-score transformation with mean of 50 ± 10 [SD] in the general US population). The Physical Activity Scale for Individuals with Physical Disabilities was used to assess the participants' physical activity (16). This scale consists of 13 items, concerning leisure time, household, and work-related activities. The questionnaire asks the number of days (during the past 7 days) and hours (per day) a respondent engaged in each activity. The total score is calculated by multiplying the average hours per day for each activity by a metabolic equivalent value associated with the intensity of the activity (range 0–182). To obtain the severity of disabilities in terms of physical independence (Activities of Daily Living: ADL), we used the Barthel Index (17). Higher scores on these instruments indicate better status in health, physical activity, or independence.
  The scores of the Barthel Index range between 0 and 100, indicating the different levels of independence: 0–20: total dependence; 21–60: severe dependence; 61–90: moderate dependence; 91–99: slight dependence; and 100: independent (18). For our analyses, we simplified groupings into three severity levels of physical disabilities: severe: 0–60; moderate: 61–90; and slight/independent: 90–100.
2. Demographic
  This section included questions on the participant's age, gender, diagnosed disability, year first having the disability, whether having a progressive disability or not, type of walking device if applicable, working status, and geographic location.
3. Experiences Related to Acquiring the Service Dog
  Participants reported their experiences in living with the current and past service dog(s) if they had lived with two or more service dogs. Items in the parenthesis were offered, and otherwise they were asked to write their answers: years of living with service dogs (less than 1 year, 1–2 years, 3–4 years, 5–7 years, 8–10 years, 11–15 years, 16–20 years, and more than 20 years) and current service dog (<6 months, 6–11 months, 1–2 years, 3–4 years, 5–7 years, 8–10 years, and > 10 years); the breed and weight of the current service dog (small: up to 22 lbs, medium: 23–40 lbs, large: over 41 lbs); the training history for the current dog (service dog training facility, private dog trainer, I trained my dog under the instruction of a service dog training facility, I trained my dog under the instruction of a private dog trainer, I trained my dog by myself, and other); whether acquiring the dog included a team–training (yes, and no); anxiety before acquiring the first service dog (none, taking care of a dog, expense for a dog, space for house, handling of a dog, team training, family members, neighborhood, finding a suitable agency, school/work, public access, housing, and other); the duration after acquiring the dog or deciding to train their own dog until the current dog started to perform the expected assisting tasks (<1 month, 1–2 months, 3–6 months, 7–11 months, >1 year, and the dog has not become to perform tasks that I require yet); the person with responsibility for supervising the current dog (mainly the service dog partner, half and half with an assisting person and the service dog partner, mainly an assisting person, and other); and the person who takes care of the dog [you, my family member(s), my friend(s), specially organized volunteer(s) for me, paid assistant(s), and other]. The question on responsibility was included because, when a person has severe disabilities, a family member may assume responsibility for the service dog's care. Also, the working environment of the current service dog was characterized (mainly inside the house, mainly outside of the house, and both inside and outside of the house).
4. Retrospective Ratings of Social and Psychological Aspects
  Participants rated any perceived changes in the following variables after acquiring their first service dog (increased, decreased, no changes): their frequencies of going to school or work, going out of the house, participating in public activities, meeting friends, making new friends, their required hours of paid and unpaid assistance, financial cost of assistance, and their psychological experiences, including self-esteem, social networks, relationships with other persons, independence, life satisfaction, social acknowledgment, stress, anxiety, loneliness, and depression. In addition, the participants were asked whether they experienced discomfort when meeting strangers outside of the house (yes, and no); for those who experienced it, they were asked to rate the extent to which they feel their discomfort was alleviated by the presence of their dog (never, rarely, occasionally, frequently, and often).
5. Burdens Experienced When Living With a Service Dog
  Participants rated whether they experienced specific burdens from living with their service dogs (not applicable; no; yes, I feel a little; yes, I feel moderately; and yes, I feel a lot); 20 individual items inquired about interactions with their dogs and other people in public. The items included: caring for daily needs, physically maintaining, expense for your dog, house cleaning, travel arrangements, responsibility for your dog, disease of your dog, adjustment period of being a partner with your dog, team training, daily training, any behavior problems, poor match between you and the dog, lack of skills as your service dog, refusal to obey certain commands, public people's petting interferes, challenges to access, unwanted attention, negative effect on your family relationship, causing asthma and/or allergic rhinitis to people, and facing the death of dog.
6. Effects of Service Dogs for Family Members
  Participants rated their family members' experiences after they acquired their service dogs: whether the frequency of their family members going out of the house increased, whether they were satisfied with the service dog, whether they relaxed more, and whether they felt burdened with taking care of the dog (no; yes, a little; yes, moderately; and yes, a lot).

### Inclusion and Exclusion Criteria

This study targeted any people who lived with service dogs. There were no exclusion criteria on the types of service dogs, the human partners' disabilities, and whether they had single or multiple disabilities. However, for this paper we only focused on people with a single category of disability (one of the following: mobility disability, psychiatric disability, or medical disability) who lived with a service dog which had a single role for the partner's disability (one of the following: mobility support, psychiatric support, or medical support). Roles of service dogs vary greatly and some dogs perform multiple roles for their human partners. People with multiple disabilities beyond the single categories may have more varied physical and mental conditions compared to people with a single category of disability. The diversity among service dogs with multiple roles and human partners with multiple disabilities may make it difficult to specify the differences among different types of service dogs. Therefore, in this study we only included service dogs with a single role living with a person with a single category of disability. People with multiple disabilities beyond a single category of disability and service dogs with multiple roles were not included in this: for example, a service dog partner who had amputation of parts of body (mobility disability) and diabetes (medical disability), a service dog partner who had cerebral palsy (mobility disability) and PTSD (psychiatric disability), and a service dog whose roles were mobility and psychiatric supports. Responses from these partners will be reported in a subsequent paper.

### Categories of Assistance Dogs and Partners for Comparisons of Groups

Firstly, the 19 men and 147 women participants were classified into three disability groups according to their diagnosed disabilities: solely mobility (e.g., spinal cord injury, rheumatism, and cerebral palsy); psychiatric (e.g., PTSD, anxiety, autism, and depression); and medical (e.g., diabetes and epilepsy) disabilities. Secondly, the service dogs were classified into three types: for mobility, psychiatric, and medical assistance.

Based on the participants' reported disabilities and types of service dogs, three categories of service dog teams were extracted, in all cases where only one type of disability was involved: partners with only an orthopedic disability(ies), living with mobility service dogs (mobility SD); partners with only psychiatric disability(ies) living with psychiatric service dogs (psychiatric SD); and partners with medical disability(ies) living with medical service dogs (medical SD). Using these three categories, we studied the following comparisons.

#### Comparison 1: Training Background of Dogs

The disabilities and experiences of the human partners with their dogs were compared as related to the dogs' specific training histories: self-trained service dogs (SelfSD: these dogs were trained by the partners themselves, or trained by partners guided by service dog facilities or private trainers), and professionally trained service dogs (ProSD: these dogs were trained by trainers within service dog facilities or private dog trainers). Typically, professionally trained service dogs are placed with their human partners when they are around 2 years old after completing training to be service dogs. Therefore, SelfSD partners who have lived with their current service dogs less than 2 years may be still in training. To assess the SelfSD partners' experiences with service dogs, participants were partitioned into those living with their SelfSD less than 2 years (immature SelfSD partners), and those living with their dogs for 2 or more years (mature SelfSD partners). Data were analyzed among ProSD (*n* = 73, 44.0% of total participants), immature SelfSD (*n* = 33, 19.9%), and mature SelfSD partners (*n* = 43, 25.9%). Data of those living with their first service dog were also separately analyzed.

#### Comparison 2: Severity of Mobility Disabilities

Here differences were assessed in responses associated with the severity of the partners' mobility disabilities. This included only people who have solely mobility disabilities and live with mobility service dogs. The severity of partners' disabilities was classified in three levels: severe (Barthel Index of 0–60, *n* = 30, 18.1%), moderate (61–90, *n* = 44, 26.5%), and slight/independent (91–100, *n* = 29, 17.5%).

#### Comparison 3: Types of Service Dogs

Differences were investigated in responses among partners with three types of service dogs: partners who have only mobility disabilities living with mobility service dogs (*n* = 103, 62.0%), partners who have only psychiatric disabilities living with psychiatric service dogs (*n* = 38, 22.9%), and partners who have only medical disabilities living with medical service dogs (*n* = 25, 15.1%).

### Statistical Analyses

Surveys with more than one third of answers missing were not included in the final analyses that included 19 male and 147 female participants. Statistical analyses included the Chi-square test, Mann-Whitney *U*-test, and Kruskal-Wallis test to investigate the differences between/among the specific groups (*p* < 0.05). When the Chi-square test was significant, adjusted standardized residuals were checked. Also, when the Kruskal-Wallis test for the comparisons among three groups were significant, the Mann-Whitney *U*-test with Bonferroni test (*p* < 0.0166) was used, to assess the differences between the groups. Only when the second test was significant was it shown in the results. The denominators differ for each analysis as some participants' answers were missing.

## Results

The results from comparisons of dogs with different training histories are presented first: Comparison 1. The results from the comparisons related to the partners' severity of mobility disabilities, and the different types of service dogs are presented next: Comparisons 2 and 3. Table 1 shows the demographic information on participants for each comparison group. A chi-squared test showed some significant associations between each comparison group and some demographic items. ProSD partners had lived longer with their disabilities than the immature SelfSD partners. ProSD partners used their wheelchairs significantly more and immature SelfSD partners used wheelchairs significantly less than expected. Mobility SD partners having severe disabilities used their wheelchairs significantly more and those with slight disabilities or independent in ADL used wheelchairs significantly less than expected. Similarly, mobility SD partners used wheelchairs significantly more and medical SD and psychiatric SD partners used wheelchairs significantly less than expected. Mobility SD partners had progressive disabilities significantly more and psychiatric SD partners had progressive disabilities significantly less than expected. The ADL scores were significantly lower for the ProSD partners than in the mature SelfSD partners. Similarly, mobility SD had significantly lower ADL scores than medical SD and psychiatric SD partners. On the other hand, the scores of the mental component summary in the SF-36 were significantly lower in the immature and mature SelfSD partners than the ProSD partners.

**Table 1:** Demographic of service dog partners.

**Comparisons**	**1. Training background** **Self/professional**				**2. Level of disabilities (mobility service dog)** **ADL scores I/II/III**				**3. Types of service dogs** **Mobility/medical/psychiatric service dogs**			
	**Pro**	**Self**			**Activity of Daily Living (ADL) Score**				**Mobility SD/only mobility disability** **(*n* = 103)**	**Medical SD/only medical disability** **(*n* = 25)**	**Psychiatric SD/only psychiatric disability** **(*n* = 38)**	***p***
	**(*n* = 73)**	**Immature** **(*n* = 33)**	**Mature** **(*n* = 43)**	***p***	**I: 0–60** **(*n* = 30)**	**II: 61–90** **(*n* = 44)**	**III: 91–100** **(*n* = 29)**	***p***				
Age (median, years old)	41–50	31–40	41–50	–	41–50	41–50	41–50	–	41–50	41–50	31–40	–
Female (%)	91.3	84.8	95.3	–	76.7	90.9	96.6	–	88.3	92.0	86.8	–
Years having disabilities (median, years)	>20	6–10	>20	a	>20	>20	16–20	–	>20	>20	11–15	–
Having progressive disabilities (%)	54.8	48.5	58.1	–	56.7	61.4	72.4	–	63.1	36.0	31.6	i
Main walking aid: wheelchair (%)	41.1	6.1	21.2	b	86.7	54.5	0.0	f	50.5	0.0	0.0	j
Employment status: paid full/part-time or self-employed (%)	34.2	30.3	39.5	–	43.3	45.5	44.8	–	44.7	56.0	28.9	–
ADL (median, score)	80	95	95	c	40	77.5	95	g	75	100	100	k
SF-36												
Physical component summary(mean ± SD)	38.1 ± 11.9	37.7 ± 13.7	36.9 ± 11.3	–	29.1 ± 7.1	34.0 ± 9.1	34.0 ± 7.0	h	32.6 ± 8.2	48.3 ± 9.7	47.2 ± 10.4	l
Mental component summary (mean ± SD)	46.5 ± 11.7	35.9 ± 13.2	39.6 ± 11.3	d	46.0 ± 10.8	47.6 ± 12.3	44.9 ± 10.6	–	46.4 ± 11.3	43.0 ± 11.3	29.6 ± 10.4	m
Physical activity score (mean ± SD)	15.3 ± 15.9	14.6 ± 15.1	11.0 ± 11.1	–	10.7 ± 9.6	16.8 ± 16.9	17.9 ± 16.9	–	15.3 ± 15.3	19.2 ± 14.4	14.4 ± 13.3	–
Size of dogs: large (over 41 lbs or 18 kgs, %)	98.6	81.8	79.1	e	90.0	95.5	79.3	—	89.3	80.0	81.6	–

*a: Pro-immature SelfSD: U = 1,721, p < 0.001, r = 0.36; immature SelfSD-mature SelfSD: U = 405, p = 0.0014, r = 0.37*.

b: X^2^(2) = 14.83; p < 0.001, Cramer's V = 0.33, R_adj_: ProSD-wheelchair (3.6); ProSD-others/no aids (−3.6); immature SelfSD-wheelchair (−3.2); immature SelfSD-others/no aids (3.2)

c: ProSD-mature SelfSD: U = 2,067, p = 0.004, r = 0.26.

d: ProSD-immature SelfSD: U = 666, p < 0.001, r = −0.36; ProSD-mature SelfSD: U = 1,058, p = 0.003, r = −0.27

e: X^2^(4) = 14.1; p = 0.007, Cramer's V = 0.22, R_adj_: ProSD-large (≥41 lbs) (3.6); ProSD-medium (40–23 lbs) (−2.6); ProSD-small (≤ 22 lbs) (−2.5); mature SelfSD-large (−2.6); mature SelfSD-medium (2.3)

f: X^2^(2) = 49.9; p < 0.001, Cramer's V = 0.55, R_adj_: I-wheelchair (5.4); I-others/no aids (−5.4); III-wheelchair (−6.3); III-others/no aids (6.3)

g: I–II: U = 0, p < 0.001, r = −0.84; I-III: U = 0, p < 0.001, r = −0.86; II–III: U = 0, p < 0.001, r = −0.84

h: I–III: U = 271, p = 0.013, r = 0.32

i: X^2^(2) = 14.0; p < 0.001, Cramer's V = 0.29, R_adj_: mobility-having progressive disability (3.7); mobility-not having progressive disability (−3.7); psychiatric-having progressive disability (−2.8); psychiatric-not having progressive disability (2.8)

j: X^2^(2) = 44.9; p < 0.001, Cramer's V = 0.61, R_adj_: mobility-wheelchair (6.7); mobility-others/no aids (−6.7); medical-wheelchair (−3.6); medical-others/no aids (3.6); psychiatric-wheelchair (−4.7); psychiatric-others/no aids (4.7)

k: Mobility-medical: U = 2,327, p < 0.001, r = 0.55; mobility-psychiatric: U = 3,349, p < 0.001, r = 0.54

l: Mobility-medical: U = 2,301, p < 0.001, r = 0.54; mobility-psychiatric: U = 3,394, p < 0.001, r = 0.56

*m: Mobility-psychiatric: U = 512, p < 0.001, r = −0.57; medical-psychiatric: U = 776, p < 0.001, r = 0.53*.

Most dogs of each group were large dogs weighing 18 kg (41 lb) or more, with members of the ProSD group living with large dogs significantly more and those in the mature SelfSD group living with large dogs significantly less than expected (Table 1).

The average duration of living with service dogs was longer for mobility SD partners [median years: I (severe): 8–10; II (moderate): 5–7; III (slight/independent): 5–7; mobility SD: 5–7; medical SD: 3–4; psychiatric SD: 3–4], but these were not statistically significant differences.

A strong majority of members of all groups had participated in team training. Among those not self-training their dogs, mobility SD partners with severe disabilities more often tended to have a team training, but this trend was not significant (I: 96.0%, *n* = 24; II: 93.5%, *n* = 29; III: 72.7%, *n* = 8, *p* = 0.063, Cramer's *V* = 0.29). There were also no differences for participating in team training among the other comparison groups (mobility SD: 91.0%; medical SD: 80.0%; psychiatric SD: 87.5%).

### Comparison 1: Training Background of Dogs (Self-Trained or Professionally Trained)

With almost equal numbers, 76 partners lived with self-trained service dogs (immature SelfSD partners: *n* = 33; mature SelfSD partners: *n* = 43), and 73 partners lived with professionally trained service dogs (ProSD). A majority of ProSD (*n* = 67) were trained by service dog training facilities and only 6 dogs were trained by private trainers.

Both immature and mature SelfSD partners reported it taking significantly longer durations for their dogs to perform expected tasks reliably compared to ProSD partners (more than 1 year–immature SelfSD: 28.6%, *n* = 6; mature SelfSD partners: 35.7%, *n* = 15; ProSD partners: 5.5%, *n* = 3, between ProSD and immature SelfSD: U = 148, *p* < 0.001, *r* = −0.57; ProSD and mature selfSD: U = 2070, *p* < 0.001, *r* = −0.71; immature SelfSD and mature SelfSD: *p* > 0.05). For this analysis, 16 ProSD partners and 11 immature SelfSD partners who had partnered with their service dog <1 year were not included, because they may have chosen “the dog has not become to perform tasks that I require yet” only because their relationship was still developing.

Mobility SD with severe disabilities acquired professionally trained service dogs significantly more and mobility SD with slight disabilities or independent in ADL trained their service dogs significantly more than expected [I: 16.7%, *n* = 5; II: 29.5%, *n* = 13; III: 62.1%, *n* = 18; *X*^2^(2) = 14.8, *p* < 0.001, Cramer's *V* = 0.39, R_adj_: I-ProSD (2.7); I-SelfSD (−2.7); III-ProSD (−3.6); III-SelfSD (3.6)]. Also, psychiatric SD partners self-trained their dogs (78.9%, *n* = 30) significantly more and mobility SD partners acquired professionally trained service dogs significantly more than expected [40.8%, *n* = 42; *X*^2^(2) = 23.6, *p* < 0.001, Cramer's *V* = 0.39, R_adj_: mobility-ProSD (3.8); mobility-SelfSD (−3.8); psychiatric-ProSD (−4.8); psychiatric-SelfSD (4.8)].

#### Perceived Changes After Acquiring Their Service Dogs

This section explains the partners' retrospective ratings of social and psychological aspects: what changes they experienced in each aspect after acquiring a service dog. It should be remembered that it is not a comparison of the partners' status before and after acquiring a service dog.

Perceived positive changes in leaving home, making new friends, independence, life satisfaction, and loneliness after acquiring their service dogs, were reported by more than 70% of partners with all training backgrounds of dogs. On the other hand, no perceived changes in school/job attendance, and hours of paid and unpaid assistance after acquiring their service dogs were reported by more than 50% of partners with all training backgrounds of dogs. Overall, perceived changes after acquiring their service dogs were similar among the partners with each training background of dogs. However, a chi-squared test showed that there were significant associations between training backgrounds of dogs and self-esteem and depression. Immature SelfSD partners perceived no change in self-esteem significantly more and mature SelfSD partners perceived increased self-esteem significantly more than expected. Also, mature SelfSD partners perceived decreased depression significantly more than expected (Table 2).

**Table 2:** Perceived changes after acquiring service dogs.

**Comparisons**	**Training background** **Self/professional**				**Level of disabilities (mobility service dog)** **ADL scores I/II/III**				**Types of service dogs** **Mobility/medical/psychiatric service dogs**			
	**Pro**	**Self**			**Activity of daily living (ADL) score**				**Mobility SD/only mobility disability** **(*n* = 97)**	**Medical SD/only medical disability** **(*n* = 25)**	**Psychiatric SD/only psychiatric disability** **(*n* = 38)**	***p***
	**(*n* = 73)**	**Immature** **(*n* = 33)**	**Mature** **(*n* = 43)**	***p***	**I: 0–60** **(*n* = 29)**	**II: 61–90** **(*n* = 40)**	**III: 91–100** **(*n* = 28)**	***p***				
School/job attendance	28.2 (4.2)	16.1 (6.5)	31.6 (2.6)	–	31.0 (0.0)	35.0 (5.0)	50.0 (7.1)	–	38.1 (4.1)	16.0 (4.0)	60.5 (7.9)	d
Leaving home	76.1 (1.4)	74.2 (3.2)	78.6 (4.8)	–	82.8 (0.0)	75.0 (2.5)	67.9 (0.0)	–	75.3 (1.0)	44.0 (12.0)	86.8 (2.6)	e
Public activities	56.3 (4.2)	48.4 (0.0)	71.4 (2.4)	–	62.1 (3.4)	65.0 (7.5)	60.7 (0.0)	–	62.9 (4.1)	56.0 (8.0)	65.8 (2.6)	–
Meeting friends	47.9 (4.2)	51.6 (3.2)	54.8 (2.4)	–	55.2 (3.4)	55.0 (0.0)	57.1 (0.0)	–	55.7 (1.0)	48.0 (4.0)	65.8 (7.9)	–
Making new friends	80.3 (0.0)	77.4 (0.0)	76.2 (2.4)	–	86.2 (0.0)	75.0 (0.0)	71.4 (0.0)	–	77.3 (0.0)	76.0 (8.0)	86.8 (2.6)	f
Hours of paid assistance	7.0 (4.2)	6.5 (3.2)	4.8 (0.0)	–	13.8 (6.9)	10.0 (5.0)	14.3 (3.6)	–	12.4 (5.2)	0.0 (0.0)	7.9 (5.3)	–
Hours of unpaid assistance	15.5 (8.5)	19.4 (3.2)	4.8 (4.8)	–	24.1 (17.2)	22.5 (2.5)	28.6 (10.7)	–	24.7 (9.3)	4.0 (4.0)	10.5 (7.9)	–
Money consumption	12.7 (42.3)	9.7 (32.3)	16.7 (35.7)	–	20.7 (20.7)	27.5 (32.5)	14.3 (21.4)	–	21.6 (25.8)	4.0 (48.0)	24.3 (29.7)	–
Self-esteem	83.1 (4.2)	67.7 (0.0)	92.9 (0.0)	a	82.8 (6.9)	85.0 (0.0)	75.0 (3.6)	–	81.4 (3.1)	68.0 (8.0)	89.5 (0.0)	–
Social network	74.6 (4.2)	58.1 (0.0)	78.6 (0.0)	–	79.3 (3.4)	82.5 (5.0)	60.7 (0.0)	–	75.3 (3.1)	68.0 (4.0)	73.7 (0.0)	–
Relationships with other persons	63.4 (1.4)	61.3 (0.0)	73.8 (0.0)	–	69.0 (3.4)	72.5 (5.0)	67.9 (0.0)	–	70.1 (3.1)	52.0 (4.0)	76.3 (0.0)	–
Independence	90.0 (1.4)	87.1 (0.0)	90.5 (0.0)	–	89.7 (3.4)	97.5 (0.0)	89.3 (0.0)	–	92.8 (1.0)	88.0 (4.0)	81.6 (0.0)	–
Life satisfaction	93.0 (0.0)	90.3 (0.0)	92.9 (0.0)	–	96.6 (3.4)	87.5 (0.0)	100.0 (0.0)	c	93.8 (1.0)	92.0 (0.0)	81.6 (0.0)	–
Social acknowledgment	74.6 (1.4)	67.7 (0.0)	81.0 (0.0)	–	82.8 (3.4)	85.0 (0.0)	71.4 (0.0)	–	80.4 (1.0)	52.0 (4.0)	73.7 (0.0)	g
Stress	74.6 (7.0)	67.7 (6.5)	78.6 (11.9)	–	62.1 (17.2)	85.0 (7.5)	85.7 (3.6)	–	78.4 (9.3)	68.0 (4.0)	73.7 (13.2)	–
Anxiety	71.8 (2.8)	67.7 (3.2)	83.3 (9.5)	–	58.6 (17.2)	77.5 (5.0)	85.7 (0.0)	–	74.2 (7.2)	64.0 (4.0)	86.8 (7.9)	–
Loneliness	74.6 (7.0)	71.0 (3.2)	81.0 (7.1)	–	75.9 (13.8)	82.5 (7.5)	78.6 (3.6)	–	79.4 (8.2)	64.0 (4.0)	76.3 (2.6)	–
Depression	59.2 (2.8)	54.8 (0.0)	81.0 (4.8)	b	62.1 (13.8)	62.5 (2.5)	71.4 (0.0)	–	64.9 (5.2)	40.0 (4.0)	78.9 (2.6)	h

*The numbers show the percentages of partners who experienced positive changes in each item. The numbers in parenthesis show percentages of partners who experienced negative changes in each item. The remaining percentages of partners which are not indicated in the table experienced no changes in each item. The p-value is not reported if > 0.05*.

a: X^2^(4) = 12.4, p = 0.014, Cramer's V = 0.21, R_adj_: immature SelfSD-no change (3.0); immature SelfSD-increased (−2.5); mature SelfSD-increased (2.1)

b: X^2^(4) = 10.4, p = 0.034, Cramer's V = 0.19, R_adj_: mature SelfSD-no change (−3.0); mature SelfSD-decreased (2.6)

c: X^2^(4) = 9.8, p = 0.044, Cramer's V = 0.22, R_adj_: II-no change (2.7); II-increased (−2.2)

d: X^2^(4) = 15.3, p = 0.004, Cramer's V = 0.22, R_adj_: medical-increased (−2.7); medical-no change (2.7); psychiatric-increased (3.0); psychiatric-no change (−3.3)

e: X^2^(4) = 18.7, p < 0.001, Cramer's V = 0.24, R_adj_: medical-increased (−3.6); medical-no change (2.6); medical-decreased (2.8); psychiatric-increased (2.2); psychiatric-no change: (−2.2)

f: X^2^(4) = 9.6, p = 0.048, Cramer's V = 0.17, R_adj_: mobility-decreased (−2.2); medical-decreased (2.5)

g: X^2^(4) = 9.5, p = 0.049, Cramer's V = 0.17, R_adj_: mobility-increased (2.2); mobility-no changes (−2.1); medical-increased (−2.8); medical-no change (2.5)

*h: X^2^(4) = 10.9, p = 0.027, Cramer's V = 0.18, R_adj_: medical-no change (2.9); medical-decreased (−2.8); psychiatric-no change (−2.0); psychiatric-decreased (2.1)*.

#### Alleviating Discomfort in Meeting Strangers

There was a significant association between training background of dogs and experienced alleviation of discomfort in meeting strangers. Immature SelfSD partners perceived no alleviation of discomfort significantly more than expected [experienced alleviation of discomfort: ProSD: 93.5%, *n* = 43; mature SelfSD: 93.9%, *n* = 31; immature SelfSD: 71.0%, *n* = 22; *X*^2^(2) = 10.3, *p* = 0.006, Cramer's *V* = 0.31, R_adj_: immature SelfSD-experienced alleviation (−3.2); immature SelfSD-no experienced alleviation (3.2)].

#### Dogs' Problem Behaviors and Burdens Experienced While Living With a Service Dog

Immature SelfSD partners experienced behavior problems with their dogs significantly more and ProSD partners experienced no behavior problems with their dogs significantly more than expected [ProSD: 15.5%, *n* = 11; immature SelfSD: 48.4%, *n* = 15; mature SelfSD: 32.5%, *n* = 13; *X*^2^(2) = 12.4, *p* = 0.002, Cramer's *V* = 0.30, R_adj_: ProSD-experienced problem behaviors (−3.2); ProSD-no behavior problems (3.2); immature SelfSD-experienced problem behaviors (3.0); immature SelfSD-no behavior problems (−3.0)].

Experienced burdens of living with a service dog were significantly associated with training backgrounds of dogs. Immature SelfSD partners experienced burdens with “negative effect on family,” “behavior problem of their dogs,” “travel arrangement,” and “unwanted attention” significantly more, and ProSD partners experienced no burdens on “travel arrangement,” “behavior problem,” and “negative effect on family” significantly more than expected (Table 3).

**Table 3:** Experienced burdens living with service dogs in each group.

	**1. Training background** **Self/professional**				**2. Level of disabilities (mobility service dog)** **ADL scores I/II/III**				**3. Types of service dogs** **Mobility/medical/psychiatric service dogs**			
**% people felt burdened at any level (% people felt burdened a lot)**	**Pro**	**Self**			**Activity of daily living (ADL) score**				**Mobility SD/only mobility disability** **(*n* = 103)**	**Medical SD/only medical disability** **(*n* = 25)**	**Psychiatric SD/only psychiatric disability** **(*n* = 38)**	***p***
	**(*n* = 73)**	**Immature** **(*n* = 33)**	**Mature** **(*n* = 43)**	***p***	**I: 0–60** **(*n* = 30)**	**II: 61–90** **(*n* = 44)**	**III: 91–100** **(*n* = 29)**	***p***				
Poor matching	0.0	0.0	0.0	–	4.5 (0.0)	0.0	5.0 (0.0)	–	2.8 (0.0)	5.3 (0.0)	4.3 (0.0)	–
Allergy/asthma	8.3 (0.0)	3.7 (0.0)	10.7 (0.0)	–	9.5 (0.0)	9.1 (0.0)	15.4 (0.0)	–	11.3 (0.0)	15.0 (0.0)	3.2 (0.0)	–
Lack of skill for assistance	8.8 (0.0)	23.1 (0.0)	12.9 (0.0)	–	8.7 (0.0)	6.0 (3.0)	13.0 (0.0)	–	8.9 (1.3)	9.5 (0.0)	26.1 (0.0)	–
Daily care	13.6 (1.5)	3.7 (0.0)	10.8 (0.0)	–	25.0 (0.0)	8.1 (0.0)	11.5 (3.8)	–	14.3 (1.1)	0.0	17.1 (0.0)	–
Responsibility	14.9 (0.0)	16.7 (3.3)	13.9 (2.8)	–	17.9 (3.6)	18.4 (2.6)	7.7 (0.0)	–	15.2 (2.2)	16.7 (0.0)	17.1 (2.9)	–
Team up	26.9 (3.0)	24.1 (0.0)	18.2 (0.0)	–	35.7 (0.0)	16.2 (2.7)	11.5 (3.8)	–	20.9 (2.2)	34.8 (0.0)	34.3 (2.9)	–
Negative effect on family	5.0 (0.0)	31.0 (0.0)	19.4 (3.2)	a	16.0 (4.0)	17.6 (0.0)	24.0 (0.0)	–	19.0 (1.2)	13.6 (4.5)	26.7 (3.3)	–
Ignore commands	13.1 (0.0)	32.0 (0.0)	22.6 (0.0)	–	25.9 (0.0)	20.7 (0.0)	20.0 (0.0)	–	22.2 (0.0)	21.7 (0.0)	25.0 (0.0)	–
Team training	31.3 (9.0)	42.1 (0.0)	25.8 (0.0)	–	38.5 (0.0)	24.2 (9.1)	25.0 (6.3)	–	29.3 (5.3)	25.0 (0.0)	38.9 (5.6)	–
Daily training	26.2 (2.4)	43.3 (0.0)	25.8 (0.0)	–	22.2 (0.0)	28.6 (2.9)	25.0 (0.0)	–	25.6 (1.2)	22.7 (0.0)	44.8 (0.0)	–
Disease	28.0 (4.0)	30.4 (0.0)	6.5 (0.0)	–	58.3 (16.7)	20.7 (6.9)	16.0 (4.0)	f	30.8 (9.0)	31.6 (0.0)	35.5 (6.5)	–
House cleaning	25.0 (1.7)	14.3 (0.0)	40.5 (2.7)	–	24.0 (0.0)	42.9 (2.9)	28.0 (0.0)	–	32.9 (1.2)	30.4 (4.3)	18.2 (0.0)	–
Behavior problems	18.6 (0.0)	53.8 (0.0)	19.4 (0.0)	b	25.0 (0.0)	31.4 (0.0)	20.8 (0.0)	–	26.5 (0.0)	40.9 (0.0)	41.9 (0.0)	–
Physical maintenance	33.9 (6.5)	34.5 (0.0)	30.6 (2.8)	–	38.5 (3.8)	41.7 (2.8)	34.6 (3.8)	–	38.6 (3.4)	29.2 (0.0)	28.6 (0.0)	–
Travel arrangement	34.4 (3.3)	66.7 (6.7)	57.1 (2.9)	c	42.3 (0.0)	38.2 (2.9)	40.7 (7.4)	–	40.2 (3.4)	52.2 (8.7)	54.5 (0.0)	–
Expense	42.4 (1.5)	38.7 (0.0)	57.9 (2.6)	–	50.0 (3.6)	43.2 (2.7)	44.4 (3.7)	–	45.7 (3.3)	41.7 (4.2)	61.1 (5.6)	–
Public access	52.3 (0.0)	50.0 (10.0)	70.3 (8.1)	d	46.4 (0.0)	45.9 (5.4)	68.0 (8.0)	–	52.2 (4.4)	66.7 (8.3)	62.9 (8.6)	–
Unwanted attention	53.7 (7.5)	70.0 (6.7)	67.6 (29.7)	e	46.4 (10.7)	60.5 (13.2)	69.2 (15.4)	–	58.7 (13.0)	52.2 (17.4)	83.3 (13.9)	g
Death	65.4 (21.2)	66.7 (25.9)	88.6 (45.7)	–	85.2 (33.3)	65.5 (34.5)	58.3 (37.5)	–	70.0 (35.0)	75.0 (20.0)	77.8 (27.8)	–
Petting by strangers	81.8 (13.6)	80.0 (30.0)	83.8 (27.0)	–	67.9 (14.3)	73.0 (21.6)	84.6 (23.1)	–	74.7 (19.8)	97.0 (13.0)	86.1 (33.3)	—

*Numbers show percentages of partners who felt burdened at any level with each item. The numbers in parenthesis show percentages of partners who felt burdened a lot with each item. The p-value is not reported if > 0.05*.

a: X^2^(6) = 16.6, p = 0.014, Cramer's V = 0.26, Radj: immature SelfSD-no (−2.8); immature SelfSD-yes, a little (3.3); pro-no (3.1); pro-yes, a little (−2.6)

b: X^2^(4) = 15.2, p = 0.004, Cramer's V = 0.26, Radj: immature SelfSD-no (−3.5); immature SelfSD-yes, a little (2.6); immature SelfSD-yes, a moderately (2.6); pro-no (2.0)

c: X^2^(6) = 15.9, p = 0.007, Cramer's V = 0.25, Radj: immature SelfSD-no (−2.3); immature SelfSD-yes, a little (2.0); mature SelfSD-yes, moderately (2.7); pro-no (3.0); pro-yes, moderately (−2.5)

d: X^2^(6) = 14.2, p = 0.027, Cramer's V = 0.23, Radj: immature SelfSD-yes, a little (−2.0); mature SelfSD-no (−1.95); pro-yes, a lot (−2.5); pro-yes, a moderately (−2.0)

e: X^2^(6) = 18.0, p = 0.006, Cramer's V = 0.26, Radj: immature SelfSD-yes, moderately (2.2); mature SelfSD-yes, a lot (3.4); pro-yes, a lot (−2.0)

f: X^2^(6) = 16.3, p = 0.012, Cramer's V = 0.40, Radj: I-yes, a lot (−3.5); I-yes, moderately (3.3)

*g: X^2^(6) = 14.7, p = 0.023, Cramer's V = 0.22, Radj: psychiatric-no (−2.8); psychiatric-yes, moderately (3.1)*.

#### Satisfaction With the Dogs and Impacts on Their Family Members

ProSD partners described that their family members were satisfied with their dogs a lot significantly more, and immature SelfSD partners described that their family members were not satisfied with their dogs or were satisfied with their dogs a little significantly more than expected [Yes (Yes, a lot): ProSD: 97.1%, *n* = 67 (81.2%, *n* = 56); immature SelfSD: 86.7%, *n* = 26 (46.7%, *n* = 14); mature SelfSD: 100%, *n* = 39 (69.2%, *n* = 27); *X*^2^(6) = 21.9, *p* = 0.001, Cramer's *V* = 0.28, R_adj_: ProSD-yes, a lot (2.8); ProSD-yes, a little (−3.2); immature SelfSD-yes, a lot (−3.2); immature SelfSD-yes, a little (2.9); immature SelfSD-no (2.7)].

### Comparisons 2 and 3: the Severity of Partners' Mobility Disabilities, and the Types of Service Dogs

#### Paid and Unpaid Assistance and Expenses

More than 50% of the partners reported no changes after acquiring a dog for the costs of paid and unpaid assistance and related expenses (Table 2). Fewer than 15% of the participants in all groups reported a decrease in paid assistance after acquiring a service dog. For unpaid assistance, slightly more partners reported a decrease, about 20%. More mobility SD partners tended to report decreased times for paid and unpaid assistance than others. But overall, no change for the assisting hours was the most common answer for all groups. Also, there were no statistically significant differences between/among the groups.

Participants indicated whether their service dogs caused an increase or decrease of expenses. The reported increased expenses and decreased expenses were in similar proportions for the mobility SD and psychiatric SD partners. On the other hand, medical SD partners reported increased expenses more often than a decrease. However, there were no statistically significant differences between/among the groups, and about half of the participants answered that there were no changes for the related expenses.

#### Participation in Society and Psychological Aspects

The frequency of school/work attendance and leaving home differed among partners with the various service dogs. Medical SD partners reported no perceived change of school/work attendance and leaving home significantly more than expected.

More than 70% of the participants in all groups answered that their dogs facilitated making new friends. Most participants reported improvements for psychological aspects after acquiring a service dog (Table 2). However, mobility SD partners with moderate disabilities reported no perceived changes in life satisfaction significantly more than expected. Furthermore, mobility SD partners perceived increased social acknowledgment significantly more than expected, while medical SD partners perceived no changes in social acknowledgment significantly more than expected. Similarly, psychiatric SD partners perceived decreased depression significantly more than expected, but medical SD partners perceived no changes in depression significantly more than expected.

#### Concerns Before Acquiring a Service Dog and Getting Teamed-Up With Their Dog

Mobility SD partners with slight disabilities or independent in ADL did not have concerns before acquiring a service dog significantly more than expected [had concern: I: 79.3%, *n* = 23; II: 75.0%, *n* = 30; III: 46.4%, *n* = 13, *X*^2^(2) = 8.6, *p* = 0.010, Cramer's *V* = 0.30, R_adj_: III-no concern (2.9); III-had concern (−2.9)]. Mobility SD partners with severe disabilities had concern on the care of a dog before acquiring a service dog significantly more than expected (I: 41.4%, *n* = 12; II: 20.0%, *n* = 8; III: 14.3%, *n* = 4, *X*^2^(2) = 6.4, *p* = 0.040, Cramer's *V* = 0.26, R_adj_: I-had concern with the care of a dog (2.5); I-no concern with the care of a dog (−2.5)]. In other items for the prior concerns, there were no differences among people with different severities of disabilities; expense of dog (I: 27.6%, 8; II: 27.5%, *n* = 11, III: 17.9%, *n* = 5), space of house (I: 10.3%, *n* = 3, II: 17.5%, *n* = 7; III: 7.1%, *n* = 2), handling of dog (I: 34.5%, *n* = 10; II: 22.5%, *n* = 9; III: 21.4%, *n* = 6), team training (I: 24.1%, *n* = 7; II: 12.5%, *n* = 5; III: 7.1%, *n* = 2), family members (I: 27.6%, 8; II: 25.0%, *n* = 10; III: 21.4%, *n* = 6), neighborhood (I: 0.0%, *n* = 0; II: 5.0%, *n* = 2; III: 3.6, *n* = 1), finding an adequate training facility (I: 24.1%, *n* = 7; II: 17.5%, *n* = 7; III: 7.1%, *n* = 2), their work and school (I: 10.3%, *n* = 3; II: 22.5%, *n* = 9; III: 17.9%, *n* = 5), public access (I: 10.3%, *n* = 3; II: 27.5%, *n* = 11; III: 32.1%, *n* = 9), housing (I: 6.9%, *n* = 2; II: 7.5%, *n* = 3; III: 3.6%, *n* = 1). Among other comparison groups, no significant differences in concerns were confirmed.

Participants reported the duration of time (<1 month, 1–2 months, 3–6 months, 7–11 months, > 1 year) until their dogs started to perform tasks they required, after the dog came from a training agency, or after the participant decided to train the dog as a service dog. Psychiatric SD partners (median: 3–6 months) took significantly longer durations than mobility SD (median: <1 month, *U* = 645, *p* < 0.001, *r* = 0.32). Among other comparison groups, no significant differences in these durations were confirmed.

#### Responsibility and Care for the Dog

Most participants had responsibility for their dogs (I: 82.8%, *n* = 24; II: 94.9%, *n* = 37; III: 96.4%, *n* = 27; mobility SD: 91.7%, *n* = 88; medical SD: 100%, *n* = 25; psychiatric SD: 89.2%, *n* = 33) and took care of them by themselves (I: 17.2%, *n* = 5; II: 50.0%, *n* = 20; III: 60.7%, *n* = 17; mobility SD: 43.3%, *n* = 42; medical SD: 72.0%, *n* = 18; psychiatric SD: 59.5%, *n* = 22). However, mobility SD with severe disabilities took care of their dogs by themselves significantly less than expected, and they more often shared the care with other people or totally depended on others for the care [*X*^2^(2) = 12.2, *p* = 0.002, Cramer's *V* = 0.36, R_adj_: I-taking care of the dog by themselves (−3.4); I-sharing the care with other people/totally depending on others for the care (3.4); III-taking care of the dog by themselves (2.2); III-sharing the care with other people/totally depending on others for the care (−2.2)].

#### Dogs' Tasks and Working Environments

The tasks performed by the service dogs differed depending on the severity of the disabilities among their mobility SD partners. Those with severe disabilities mentioned the following tasks significantly more than expected: barking on command [I: 20.7%, *n* = 6; II: 10.0%, *n* = 4; III: 0.0%, *X*^2^(2) = 6.6, *p* = 0.037, Cramer's *V* = 0.26, R_adj_: I-yes (means the SD performed the task) (2.2); I-no (means the SD did not perform the task) (−2.2); III-yes (−2.1); III-no (2.1)], calling someone [I: 31.0%, *n* = 9; II: 15.0%, *n* = 6; III: 3.6%, *n* = 1, *X*^2^(2) = 7.9, *p* = 0.019, Cramer's *V* = 0.29, R_adj_: I-yes (2.5); I-no (−2.5); III-yes (−2.2); III-no (2.2)], helping to take off clothes [I: 37.9%, *n* = 11; II: 30.0%, *n* = 12; III: 3.6%, *n* = 1, *X*^2^(2) = 10.0, *p* = 0.007, Cramer's *V* = 0.32, R_adj_: I-yes (2.0); I-no (−2.0); III-yes (−3.1); III-no (3.1)], opening/closing doors [I: 75.9%, *n* = 22; II: 50.0%, *n* = 20; III: 21.4%, *n* = 6, *X*^2^(2) = 16.9, *p* < 0.001, Cramer's *V* = 0.42, R_adj_: I-yes (3.4); I-no (−3.4); III-yes (−3.5); III-no (3.5)], retrieving dropped objects [I: 89.7%, *n* = 26; II: 95.0%, *n* = 38; III: 75.0%, *n* = 21, *X*^2^(2) = 6.2, *p* = 0.044, Cramer's *V* = 0.25, R_adj_: III-yes (−2.4); III-no (2.4)], and retrieving objects out of reach [I: 72.4%, *n* = 21; II: 55.0%, *n* = 22; III: 35.7%, *n* = 10, *X*^2^(2) = 7.7, *p* = 0.021, Cramer's *V* = 0.28, R_adj_: I-yes (2.3); I-no (−2.3); III-yes (−2.4); III-no (2.4)]. In contrast, mobility SD partners with slight disabilities or independent in ADL chose the following tasks significantly more than expected: helping to stand up [I: 6.9%, *n* = 2; II: 35.0%, *n* = 14; III: 53.6%, *n* = 15, *X*^2^(2) = 14.6, *p* < 0.001, Cramer's *V* = 0.39, R_adj_: I-yes (−3.5); I-no (3.5); III-yes (2.9); III-no (−2.9)], and supporting balance [I: 13.8%, *n* = 4; II: 42.5%, *n* = 17; III: 89.3%, *n* = 25, *X*^2^(2) = 33.2, *p* < 0.001, Cramer's *V* = 0.59, R_adj_: I-yes (−4.3); I-no (4.3); III-yes (5.3); III-no (−5.3)]. Furthermore, mobility partners with moderate disabilities chose pulling my wheelchair significantly more than expected [I: 6.9%, *n* = 2; II: 25.0%, *n* = 10; III: 7.1%, *n* = 2, *X*^2^(2) = 6.2, *p* = 0.046, Cramer's *V* = 0.25, R_adj_: II-yes (2.5); II-no (−2.5)]. The tasks of carrying objects (I: 48.3%, *n* = 14; II: 45.0%, *n* = 18; III: 46.4%, *n* = 13) were frequently chosen by each group of mobility SD partners.

Medical SD partners frequently mentioned tasks of: alerting to a problem with blood sugar (64.0%, *n* = 16), alerting to seizures, calling someone, and retrieving objects out of reach (each 20.0%, *n* = 5). Psychiatric SD partners often reported the following tasks: giving tactile stimulation for psychiatric symptoms (83.8%, *n* = 31), alerting to incipient anxiety or panic attack (78.4%, *n* = 29), reminding to take medication, and helping to stand up (each 24.3%, *n* = 9).

Concerning their dogs' working environments, most participants answered that tasks both inside and outside of the house were important (I: 93.1%, *n* = 27; II: 90.0%, *n* = 36; III: 82.1%, *n* = 23; mobility SD: 88.7%, *n* = 86; medical SD: 88.0%, *n* = 22; psychiatric SD: 76.3%, *n* = 29). Very few participants considered only the tasks inside of the house as being important (I: 6.9%, *n* = 2; II: 5.0%, *n* = 2; III: 3.6%, *n* = 1; mobility SD: 5.2%, *n* = 5; medical SD: 4.0%, *n* = 1; psychiatric SD: 0.0%).

#### Alleviating Discomfort in Meeting Strangers

More than half of the participants in all groups but medical SD partners experienced discomfort when they meet strangers outside of the house (I: 58.6%, *n* = 17; II: 72.5%, *n* = 29; III: 60.7%, *n* = 17). Psychiatric SD partners reported this significantly more than expected [mobility SD: 64.9%, *n* = 63; medical SD: 48.0%, *n* = 12; psychiatric SD: 100%, *n* = 37, *X*^2^(2) = 23.0, *p* < 0.001, Cramer's *V* = 0.38, R_adj_: medical-yes (−2.7); medical-no (2.7); psychiatric-yes (4.5); psychiatric-no (−4.5)]. More than half of the people who experienced discomfort felt it was alleviated frequently or often with the presence of their dog (I: 82.4%, *n* = 14; II: 79.3%, *n* = 23; III: 76.5%, *n* = 13; mobility SD: 51.5%, *n* = 50; medical SD: 58.3%, *n* = 7; psychiatric SD: 89.2%, *n* = 33). There were no statistically significant differences about this among/between the groups.

#### Dogs' Problem Behaviors and Burdens Experienced When Living With a Service Dog

The problem behaviors of dogs experienced did not differ between or among the groups. Those having problem behaviors of their dogs at the time of the survey were: I: 6.9%, *n* = 2; II: 12.5%, *n* = 5; III: 10.7%, *n* = 3; mobility SD: 10.3%, *n* = 10; medical SD: 16.0%, *n* = 4; psychiatric SD: 13.5%, *n* = 5; and earlier had experienced problem behaviors: I: 17.2%, *n* = 5; II: 17.5%, *n* = 7; III: 10.7%, *n* = 3; mobility SD: 15.5%, *n* = 15; medical SD: 20.0%, *n* = 5; psychiatric SD: 27.0%, *n* = 10.

Table 3 summarizes the burdens each group reported, listed from least to most frequently reported. Mobility SD partners with severe disabilities felt burdened moderately with diseases of their dogs significantly more than expected. Psychiatric SD partners felt burdened with unwanted attention from others significantly more than expected. No statistically significant differences were seen between/among other comparison groups.

#### Satisfaction With the Dog and Impacts on Their Family Members

Participants rated their levels of satisfaction with their dogs, selecting from 0, 25, 50, 75, and 100%. Most participants answered that they were satisfied with their dog at 100% (I: 86.2%, *n* = 25; II: 87.5%, *n* = 35; III: 89.3%, *n* = 25; mobility SD: 87.6%, *n* = 85; medical SD: 83.3%, *n* = 20; psychiatric SD: 77.8%, *n* = 28). No statistically significant differences were seen between/among the groups.

The participants rated the impacts of their dogs for family members. Concerning the family's satisfaction with their dogs, most of the participants answered that their family members felt somewhat satisfied with their dogs, which was most commonly reported by the mobility SD partners with severe disabilities (I: 100%, *n* = 29; II: 95.0%, *n* = 38; III: 96.4%, *n* = 27; mobility SD: 96.9%, *n* = 94; medical SD: 95.8%, *n* = 23; psychiatric SD: 88.9%, *n* = 32). For the family's burden on taking care of dogs, some of the participants indicated that their family members felt somewhat burdened; mobility SD partners with severe disabilities also most commonly reported this burden (I: 31.0%, *n* = 9; II: 25.0%, *n* = 10; III: 21.4%, *n* = 6; mobility SD: 25.8%, *n* = 25; medical SD: 16.7%, *n* = 4; psychiatric SD: 19.4%, *n* = 7). Furthermore, more than 60% of the participants answered that their family members relaxed more than before they acquired the dog: again most often reported by the mobility SD partners with severe disabilities (I: 89.7%, *n* = 26; II: 77.5%, *n* = 31; III: 67.9%, *n* = 19; mobility SD: 78.4%, *n* = 76; medical SD: 87.5%, *n* = 21; psychiatric SD: 77.8%, *n* = 28). About the frequency of family going out of the house, some of the participants reported that their family members went out of the house more frequently than before: also most often reported by the mobility SD partners with severe disabilities (I: 55.2%, *n* = 16; II: 32.5%, *n* = 13; III: 39.3%, *n* = 11; mobility SD: 41.2%, *n* = 40; medical SD: 29.2%, *n* = 7; psychiatric SD: 52.8%, *n* = 19). However, there were no statistically significant differences between/among the groups.

## Discussion

Clarifying the expanded roles of service dogs in the U.S., service dog partners with various backgrounds and disabilities, dogs' training backgrounds, and types of service dogs described their experiences. Results on comparisons of dogs with different training backgrounds are discussed first (Comparison 1), and then results from the comparisons related to the person's severity of mobility disabilities and the different types of service dogs (Comparisons 2 and 3).

### Comparison 1: Training Background of Dogs (Self-Trained and Professionally Trained)

Partners' experiences with service dogs differed depending on the training backgrounds: training by their partners, or professional trainers. The time required for dogs to start performing the expected tasks was longer with self-trained service dogs (SelfSD) than professionally trained service dogs (ProSD). This was a natural result of ProSD being placed with their human partners after they already had completed their training as service dogs.

#### Demographic Differences

The demographics differed between the SelfSD and ProSD; SelfSD partners had more psychiatric disabilities and fewer mobility disabilities compared to ProSD partners. Thus, the SelfSD partners had higher ADL scores, but lower scores on the mental component summary in the SF-36 than the ProSD partners. People with psychiatric disorders may prefer to train their service dogs by themselves, as was recommended by Dr. Joan Esnayra, the founder of the Psychiatric Service Dog Society. This organization formerly provided useful information about training and utilization of psychiatric service dogs. She presented some advantages of self-training one's own service dog: people can choose a favorite breed and strengthen a relationship by raising the dog from a puppy; and dogs learn the partners' physical and behavioral characteristics through this process (19). However, there are also some disadvantages of training one's own service dog. For example, the failure rate of service dogs with self-training is very high. Another concern is that it is hard to decide that one's own dog is not suitable as a service dog because of being too emotionally invested to make a decision (20). But in some cases, people may decide to train their pet dogs after they have demonstrated a suitable temperament or even exhibited helpful behaviors for supporting disabilities. SelfSD included more small dogs compared to ProSD; service dog training facilities often use large breeds like Labrador Retrievers and Golden Retrievers. Furthermore, the self-training was more frequent in mobility SD partners with slight disabilities or independent in ADL than mobility SD partners with severe and moderate disabilities. As discussed later, service dogs that are not fully trained are more likely to show behavior problems; thus, the partners of self-trained dogs experienced more burdens with behavior problems than trained service dogs. Therefore, people with more severe physical disabilities may not choose self-training.

#### Perceived Changes After Acquiring Their Service Dogs, Including Alleviating Discomfort in Meeting Strangers

A majority of partners in the three groups experienced positive changes in leaving home, making new friends, independence, life satisfaction, and loneliness after they acquired their service dogs, indicating that the training background of service dogs does not preclude the experienced benefits for service dog partners. However, although mature SelfSD partners experienced increased self-esteem significantly more, immature SelfSD partners experienced no changes significantly more than expected. Furthermore, immature SelfSD partners experienced less benefit of their dogs alleviating discomfort in meeting strangers compared with ProSD and mature SelfSD partners. Therefore, more positive changes can be acquired after service dogs are fully trained. Marshall (19) also reported that the number of diagnosis-specific tasks performed by psychiatric service dogs was associated with significantly decreased partners' use of psychiatric medications for partners with major depressive disorder and PTSD. Although many studies have shown psychological benefits of service dogs (5, 8, 21–26), it was not clear whether these psychosocial benefits are due specifically to the dogs' assistance as service dogs, or whether companion animals would have similar effects. These results of less benefit with immature SelfSD suggest that companion animals provide fewer psychosocial benefits than well-trained service dogs.

#### Dogs' Problem Behaviors and Burdens of Living With a Service Dog

Consistently, ProSD partners experienced significantly less burdens compared to immature and/or mature SelfSD partners with behavior problems, travel arrangement, unwanted attention, and negative effects on family members. These different levels of perceived burdens may result from SelfSD not being fully trained yet, so there may have been some difficulties in handling the less-trained dogs. This indicates that SelfSD partners experienced more burdens in the first few years, whereas ProSD partners avoided some of these burdens by receiving fully-trained service dogs. Also, it was shown that ProSD partners lived with large dogs more often than the immature and mature SelfSD partners. Traditional service dogs are usually large breeds, like Labrador Retrievers and Golden Retrievers; many large service dog training facilities use these breeds. Members of the public may look at smaller SD skeptically because of the recent increase in fake service dogs. Awareness of fraudulent service dogs may cause difficulties when SelfSD partners take their dogs in public settings.

#### Satisfaction With the Dogs and Impacts on Their Family Members

ProSD partners reported their family members as satisfied with their service dogs significantly more than expected. Similar to the partners' experienced benefits, family members experienced less benefit when their SDs were not fully trained.

### Comparisons 2 and 3: The Severity of Partners' Mobility Disabilities, and the Types of Service Dogs

Overall, the various categories of service dog partners experienced physical, psychological, and social benefits as reported previously by traditional service dog partners. The common benefits shared by the groups are discussed first, and then the differences among the groups.

#### Paid/Unpaid Assistance and Expenses

A few studies have examined changes in expenses associated with acquiring service dogs (5, 27); however, the previously reported results are inconsistent. In the randomized clinical trial of Allen and Blascovich, they reported that the presence of a service dog was associated with a decrease of approximately 60 bi-weekly paid assistance hours, 12 months following the acquisition of the service dog. They calculated that the annual reduction of expense would be about $10,000 when the hourly expense was estimated at $8 (27). A retrospective study of Fairman and Huebner found that paid assistance declined by ~2 h per week after acquiring the service dog, for a reduction of $600 per year (5). Other papers reported that acquiring service dogs increased the financial burden with expenses for the dogs (4, 8). The report by Allen and Blascovich contrasts with the other studies that reported results similar to ours: service dog partners experienced only a small decrease in paid assistance hours after acquiring their dogs.

Interestingly, although participants reported some small reductions of paid/unpaid assisting hours, about half answered that there were no overall changes in expenses when they considered the total combined expenses for paid assistance and dogs. This suggests that the expenses for the dog were somehow balanced or compensated by a reduction in assistance costs. One explanation for some reduced assistance could be increased independence of the service dog partners leading to increased attendance at work as mentioned later. Mobility SD and psychiatric SD partners reported an increase in attending their school/work more often than medical SD partners; similarly among mobility SD and psychiatric SD partners, the reported reduction in total expenses was slightly greater than what medical SD partners reported. This may explain the reduced assistance compensating for the expenses for dogs, or even a reduction of expenses in total. Another possibility is that family members had increased free time caused by the reduction of unpaid assisting hours. The family members then may have been more able to go to work using their increased free time. This indicates that the financial benefits associated with having service dogs may relate to increased employment or working time rather than decreased paid assistance time.

#### Participation in Society and Psychological Benefits

More than 70% of participants appreciated as a benefit their service dog facilitating them in making new friends. Increased participation in society and interactions with others after the acquisition of their dog were frequently reported; this is consistent with previous studies on assistance dogs (4–9). Individuals who have adequate social relationships, including three major components of a high degree of integration in social networks, social interactions that are intended to be supportive, and beliefs and perceptions of support availability held by the individual, are reported to enjoy a 50% greater likelihood of survival compared to those with poor or insufficient social relationships (28). Service dogs acting as a lubricant of social relationships over time may provide a great impact on the health of people living with them.

It is worth mentioning that 38.1% of mobility SD partners and 60.5% of psychiatric SD partners reported that the frequency of them attending to their school/work increased after the acquisition of their service dog. Reviewing the benefits of assistance dogs, Sachs-Ericsson et al. reported a trend for increased employment among assistance dog partners (23). While our survey did not specify new vs. ongoing employment, our results show an impact of assistance dogs in facilitating the partners going to school/work more frequently.

The psychological benefits experienced by each type of service dog partner were consistent with the benefits reported by traditional mobility service dog partners (5, 8, 9). For the participants who had felt discomfort when meeting others in public, the presence of their dogs helped to alleviate such feelings. All 37 participants with psychiatric service dogs experienced discomfort when meeting people in public; the dog alleviated this discomfort for almost all (*n* = 33), but a strong majority (*n* = 30) still felt burdened by unwanted attention. People with disabilities experience stigma related to their disabilities, and the negative public attitudes for their disabilities can add burdens in their daily lives, making it difficult for them to interact with others and/or participate in social activities (29, 30). Dogs provide the robust benefit of facilitating social interactions for their handlers, such as increased conversations with strangers and positive reactions from others (31). In addition, interactions with dogs have been shown experimentally to reduce the handler's cortisol and increase oxytocin, suggesting that the interactions decreased stress (32, 33). In stressful situations, people with dogs were reported to experience lower anxiety and less negative affect as compared to those without dogs (34). These dogs' layers of benefits—changing the people's reactions in positive ways, alleviating perceived stress psychologically and physiologically—would help in diminishing/decreasing the discomfort when meeting others in public.

#### Impacts for Family Members

The results indicated that the family members of service dog partners also benefited from the dogs. More than half of the participants answered that their family member went out of the house more frequently than prior to them acquiring the dog. The introduction of a service dog also led to more relaxation for their family members. These benefits are consistent with previous studies based on traditional service dogs (25, 35) and other types of assistance dogs (36) and may reflect some general characteristics of service dogs. Firstly, in emergencies, service dogs operate as a life-line for their partners with disabilities. Some service dogs were trained to call someone, retrieve objects, such as a cell phone that is out of reach, and bark on command, and these special commands were used more frequently by the mobility SD partners with severe disabilities than mobility SD partners with slight or independent in ADL and moderate disabilities. Family members of persons with severe disabilities can gain a secure feeling, knowing that the dogs can help their family members with disabilities when in need, allowing their family members then to leave home easily without having major concerns. Secondly, the increased independence the dogs provide participants would decrease the requirement for unpaid assistance and thus increase the free time of their family members. Thirdly, the acquisition of a dog would naturally have increased the frequency of going outside: dogs need exercise and outdoor toileting. Going outdoors may especially increase for the family members who share the care of the dog with the partners who have disabilities. The mobility SD partners with severe disabilities reported these benefits for family members at the highest level among the groups. The benefits of service dogs for family members may differ, reflecting the severity of the partners' disabilities and how much assistance they require from their family members.

Taking care of service dogs is a negative burden when acquiring service dogs. Davis et al. (4) showed that the family members spent 6.2 h for the dogs' care weekly; 25% of the participants felt burdened by this time expenditure. Mobility SD partners with severe disabilities more frequently than other groups reported that their family members felt the burden of caring for the dogs. Partners with severe disabilities perhaps more often share the care of dogs with others, or even totally depend on others, as compared with participants in other groups. People with severe disabilities in particular need good support and understanding from others.

The following sections discuss results where there were significant differences between/among the groups.

#### Levels of Disabilities

The mobility SD partners with slight disabilities or independent in ADL trained their dogs by themselves more often than the mobility SD partners with moderate and severe disabilities. Those with more independence may choose to train their dogs by themselves rather than being delayed until a suitable dog is available.

Mobility SD partners with severe disabilities had greater concerns than others about service dogs prior to acquiring their dogs. Providing care for the dogs was a primary concern reported by more than half of them. In a study of Japanese people who had visual, hearing, and orthopedic disabilities and did not live with assistance dogs, the perceptions toward assistance dogs also focused on the concern of care for dogs, particularly by people with visual and orthopedic disabilities more often than people with hearing disabilities (36). The concern for care of the dogs would be a natural reaction for people who have disabilities that restrict their mobility. However, in this study the mobility SD partners with severe disabilities who actually felt burdened by the daily care for a dog was about 25%; this percentage was less than those who had been concerned about the care (52%). The mobility SD partners with severe disabilities often acquired professionally trained service dogs, and then shared the care of the dog with others or totally depended on others more often than other groups. Finding enough support and establishing an effective process for care would have reduced the burden of care for the dogs. The mobility SD partners with severe disabilities experienced moderate burdens with disease of dogs significantly more than expected. When dogs get sick, they may not be able to perform tasks their partners require. Instead, more care for the dogs may be necessary. For mobility SD partners with severe disabilities, who often share the care of their dogs with others or totally depend on others, such situations may be more challenging.

#### Types of Service Dogs

Psychiatric SD partners trained their dogs by themselves more often than mobility SD and medical SD partners. The psychiatric SD took longer to perform the expected tasks effectively compared to mobility SD; this is expected because more psychiatric SD partners trained their dogs by themselves.

Psychiatric SD partners most often reported their dogs decreasing their discomfort when meeting others. Various types of symptoms occur with psychiatric disorders, including, for some people, feeling anxiety when they interact with others, such as social anxiety disorder that is commonly reported (37).

In the last decade, the U.S. experienced an increase of Emotional Support Animals (ESAs) that support people with psychiatric disabilities but are not required to be trained to provide tasks (11). Some of the benefits of dogs reported by the psychiatric SD partners might also occur for people living with ESAs. However, psychiatric SD partners most commonly reported the perceived burden of unwanted attention from others. For some people who have challenges in interacting with others, the dog may help to alleviate their discomfort, but at the same time the dog can also increase the interaction with others, including causing unwanted attention. It may be important for people who consider having a psychiatric SD or ESA to understand this attention caused by the presence of a dog that can be experienced as either a benefit or a burden by the partner.

The experienced improvements in social activities, including the frequency of going to school/work and leaving home, after the acquisition of service dogs, were the lowest among the medical SD partners compared to mobility SD and psychiatric SD partners. Although mobility SD support the mobility of the partners and psychiatric SD decrease the discomfort of the partners interacting with others (both of which directly relate to improving the outdoor activities), the tasks performed by medical SD are not specifically related to going out of the house. Also, the disabilities of medical SD partners previously may not have prevented them from leaving their homes as much as the disabilities of mobility SD and psychiatric SD partners. Therefore, although the survey did not track new vs. ongoing employment, medical SD partners already may have been engaged in school/work and leaving home even before acquiring their service dogs, as compared with mobility SD and psychiatric SD partners.

## Conclusions

This study investigated the experiences of service dog partners who have various types and severities of disabilities and live with service dogs filling different roles. Participants with disabilities reported benefits of service dogs for themselves and their family members similar to those reported by the previous studies focusing on traditional service dogs. The basic benefits were consistent across the varied types of partners' disabilities and service dogs. However, the degrees of the benefits, concerns, and burdens slightly differed among/between the groups. In addition, the comparisons among dogs with different training histories showed that the experiences with service dogs differed greatly between ProSD partners and SelfSD partners, especially when their dogs were not fully trained. Therefore, a personalized assessment and plan is required to maximize the benefits and minimize the burdens and concerns of living with service dogs based on each person's disabilities and situation, and the potential outcomes reported in this study.

Since participation in this study was voluntary, people with positive experiences may have been more likely to participate. In addition, the retrospective answers may not accurately reflect their actual experiences with their service dogs. For greater understanding and objective outcomes regarding the new types of service dogs, a prospective study focusing on each specific population may be required.

## Data Availability

All datasets generated for this study are included in the manuscript.

## Ethics Statement

The University of California Davis Institutional Review Board Protocol #340095-2 approved the study. Participation was anonymous and voluntary, therefore no written informed consent was given. Participants will not be informed individually about the accepted publication, as this study was conducted anonymously.

## Author Contributions

MY and LH, the two authors, equally contributed to study conception, design of the questionnaire, and overall study design. MY mainly worked to acquire and analyze the data, and drafted the article. LH offered critical and constructive advice throughout the data collection and drafting the article.

### Conflict of Interest Statement

The authors declare that the research was conducted in the absence of any commercial or financial relationships that could be construed as a potential conflict of interest.

## Abbreviations

ADI: Assistance Dogs International
ADL: Activities of Daily Living
MCS: mental component summary
SD: service dogs
PCS: physical component summary
ProSD: professionally trained service dogs
PTSD: posttraumatic stress disorder
SelfSD: self-trained service dogs.
